# ColXV promotes adipocyte differentiation via inhibiting DNA methylation and cAMP/PKA pathway in mice

**DOI:** 10.18632/oncotarget.18550

**Published:** 2017-06-16

**Authors:** Guannv Liu, Meihang Li, Yatao Xu, Song Wu, Muhammad Saeed, Chao Sun

**Affiliations:** ^1^ College of Animal Science and Technology, Northwest A and F University, Yangling, Shaanxi, 712100, China

**Keywords:** ColXV, adipocyte differentiation, CREB, methylation, cAMP/PKA pathway

## Abstract

Extracellular matrix (ECM), as an essential component of adipose tissue, not only provides mechanical support for adipocyte growth, but also participates in ECM-adipocyte communication via various secreted proteins, including highly enriched collagens. Collagen XV (ColXV) is a secreted non-fibrillar collagen within ECM Basement Membrane (BM) zones and well recognized as a tumor suppressor. However, the role of ColXV in adipose tissue is still unknown. In this study, high fat diet (HFD) fed mice were used as obese model, in which we deeply investigated the interaction between ColXV and adipocyte differentiation or adipose metabolism. We found great elevated ColXV expression and positive effect of ColXV on lipid deposition during adipocyte differentiation or obesity both *in vitro* and *in vivo*. cAMP response element binding protein (CREB) is a cellular transcription factor that can inhibit adipogenesis and promote lipolysis. Here we proposed ColXV as a newly discovered downstream gene of CREB. We further proved that CREB can repress adipocyte differentiation and enhance lipolysis by negatively regulating ColXV transcription. Mechanistic studies showed ColXV enhanced adipocyte differentiation and lipid deposition through reducing its DNA methylation and repressing the cAMP/PKA signaling pathway. Collectively, our study identified ColXV as a novel downstream gene for CREB and could promote adipocyte differentiation, inhibit lipolysis through repressing cAMP/PKA signaling pathway and positively regulating adipogenic markers expressions by repressing the activity of maintenance methyltransferase Dnmt1. Our data discovered a novel role of ColXV in adipocyte differentiation and provide insight into obesity and related metabolic diseases.

## INTRODUCTION

The increasing obesity and obesity-related diseases such as insulin resistance and type II diabetes are closely associated with excessive fat deposition [1–3]. Adipose tissues, which mainly consist of adipocytes, are important in balancing systemic energy levels. Two general classes of adipose tissues are found in mammals: white and brown. White adipose tissue (WAT) stores energy in the form of fatty acids in response to systemic demands, while brown adipose tissue (BAT) burns substrates, including fatty acids and glucose, to produce heat in response to various stimuli. Adipose tissue grows through hyperplasia and/or hypertrophy, which results in increasing adipocytes number and differentiating into fat-laden adipocytes. CCAAT/enhancer-binding proteins (C/EBPs) and peroxisome proliferator activated receptor γ (PPARγ) are considered as central engine for adipocyte differentiation [4]. Cyclic adenosine monophosphate (cAMP) and cAMP-dependent protein kinase A (PKA) signaling pathway are involved in lipogenesis and lipolysis process. Studies implicated activated PKA directly phosphorylates the transcription factor cyclic AMP responsive element binding protein (CREB), which promotes the expressions of lipolysis and thermogenic genes such as peroxisome proliferator γ-activated receptor coactivator 1-α (Pgc-1α) and uncoupling protein 1 (Ucp1) in adipocytes [5, 6].

Extracellular matrix (ECM) is a non-cellular three-dimensional macromolecular network composed of collagens, proteoglycans/glycosaminoglycans, elastin, fibronectin, laminins, and several other glycoproteins [7]. ECM located in the basement membrane of adipose tissues, are involved in adipose development, expansion and metabolism, even obesity and insulin resistance [8–10]. Collagen XV (ColXV, encode by Col15α1 gene), one member of collagen family, belongs to the category of MULTIPLEXINs/endostatin-producing collagens together with collagen XVIII (ColXVIII) [11, 12]. With similar structure that a central triple helical collagenous domain is interrupted by several non-collagenous (NC) domains, MULTIPLEXINs function similarly on angiogenesis, inflammation, tumorigenesis and adipose metabolism [13–15]. Studies show that ColXVIII promotes lipid deposition in adipose tissue and also causes hypertriglyceridemia in mice and human [16–18]. ColXV was primarily recognized as an important tumor suppressor [19], but was also found highly expressed in adipocytes and adipose tissues [20, 21]. However, the function of ColXV in adipocyte differentiation and its molecular mechanism have not been established.

In present study, we found that ColXV expression was increased during adipocyte differentiation and also accentuated in adipose tissues of HFD fed mice. ColXV further accelerated lipid deposition in obese mice. ColXV strengthened adipocyte differentiation and weakened lipolysis in adipocytes. Moreover, we found ColXV promoted adipocyte differentiation through negative CREB transcription, DNA methylation inhibition and inhibiting cAMP/PKA signal pathway. Our findings illustrate a novel function of ColXV in adipocyte differentiation and energy balance and ColXV may also serve as a potential drug target for obesity related diseases.

## RESULTS

### ColXV expression was increased in obese adipose tissues and mature adipocytes

**Figure 1:**
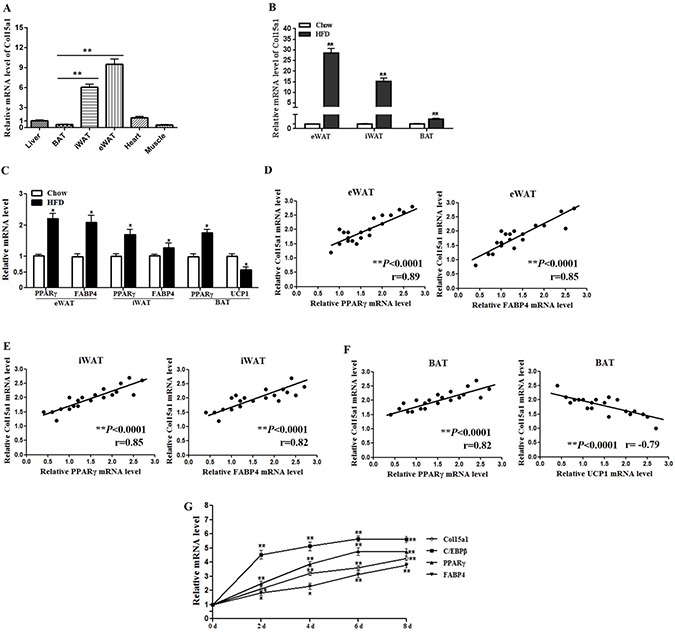
ColXV expression was increased in obese adipose tissues and mature adipocytes (**A**) Male mice of 8–10 weeks old fed with chow diet were sacrificed. ColXV mRNA level was detected in liver, BAT, iWAT, eWAT, heart and muscle (*n* = 6). To detect ColXV mRNA (**B**) and other adipogenic markers mRNA levels (**C**) in adipose tissues of obese individuals, mice were fed with HFD for 9 weeks before isolating iWAT, eWAT and BAT. Chow diet feeding mice were used as control (*n* = 8). Correlation between mRNA expression of adipogenic markers on the x-axis and mRNA expression of ColXV on the y-axis in eWAT (**D**) iWAT (**E**) and BAT (**F**) (*n* = 3). (**G**) Pre-adipocytes were acquired from four-week-old lean mice and induced into mature adipocytes. Relative mRNA levels of ColXV and adipogenic markers were detected in different time points during adipocyte differentiation (*n* = 6). Data represent the mean ± SEM of three independent experiments. *P < 0.05, ^*^P < 0.01.

We firstly detected the expression profile of ColXV in mice tissues and organs. Result showed ColXV mRNA level was higher in adipose tissues, compared with the expressions in liver, heart and muscle (Figure 1A). Among different adipose tissues, ColXV expressions in inguinal white adipose tissue (iWAT) and epididymis white adipose tissue (eWAT) were significantly higher than that in interscapular brown adipose tissue (BAT) (Figure 1A). To determine whether high fat diet (HFD)-induced obesity impacts ColXV expression, mice were fed with HFD (60 kcal% fat) or chow diet (10 kcal% fat). Higher mouse body weight and food intake were observed in mice with HFD feeding for 9 weeks (Supplementary Figure 1A and 1B). Followed by elevated adipogenic markers PPARγ, FABP4 and decreased thermogenic gene UCP1 in HFD fed mice (Figure 1C), ColXV mRNA level was robustly increased in mouse BAT, iWAT and eWAT (Figure 1B). ColXV expression was positively correlated with PPARγ and FABP4 in iWAT and eWAT, but negatively correlated with UCP1 in BAT (Figure 1D–1F). In primary cultured adipocytes, along with the elevation of adipogenic markers, ColXV mRNA level was increased during adipocyte differentiation (Figure 1G). Overall, ColXV is highly increased in obese adipose tissues and mature adipocytes, which implicate ColXV may contribute to adipocyte differentiation and adipose metabolism.

### ColXV accelerated lipid deposition in obese mice

**Figure 2:**
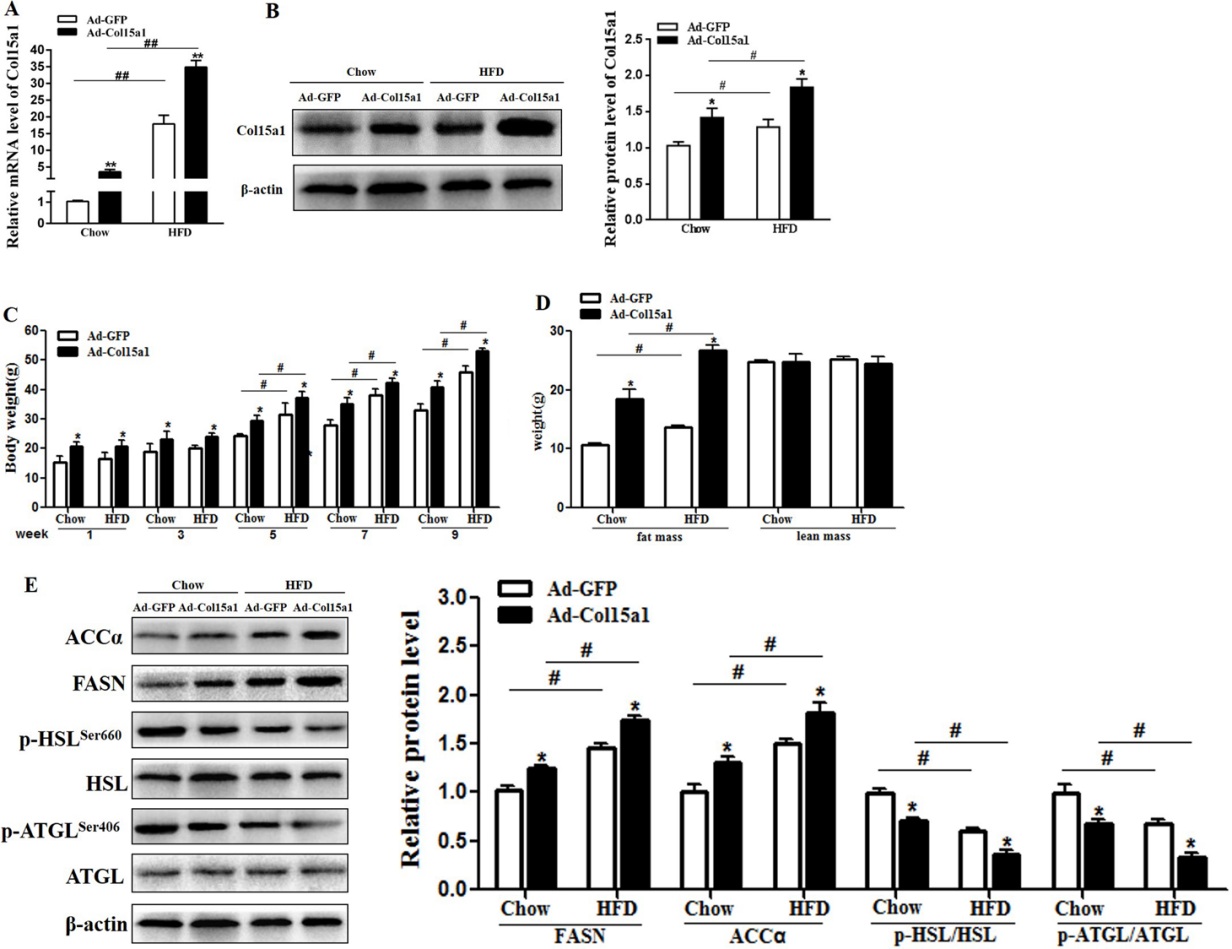
ColXV accelerated lipid deposition in obese mice Mice fed with chow and HFD diet were intraperitoneal injected with Ad-Col15α1 or Ad-GFP, then ColXV mRNA level (**A**) and protein level (**B**) were detected in eWAT of mice. Body weight (**C**) and body mass (**D**) were monitored each week. (**E**) Protein levels of ACC, FASN and phosphorylation level of HSL and ATGL were also detected in mice eWAT (*n* = 6). Data represent the mean ± SEM of three repeats. ^*#^*P* < 0.05, ^**##^*P* < 0.01.

To investigate the effects of ColXV on obesity, we intraperitoneally injected ColXV overexpressed adenovirus vector into chow and HFD fed mice. We noted that ColXV mRNA and protein expressions were significantly increased after ColXV vector injection in both chow and HFD fed mice as expected (Figure 2A and 2B), and it expressed specifically in adipose tissues (Supplementary Figure 2A). HFD fed mice showed elevated body weight and food intake relative to chow diet mice after 5 weeks, which were both strengthened in ColXV overexpressed group (Figure 2C and Supplementary Figure 2B). Then we analyzed body composition and found Col XV overexpression had a similar effect with HFD feeding, both increased the fat mass, but no effects on lean mass (Figure 2D). ColXV overexpression increased serum lipids like triglyceride (TG), total cholesterol (TC) and low-density lipoprotein (LDL-C), but decreased high density lipoprotein (HDL-C), more obvious in HFD mice (Supplementary Figure 2C). In HFD fed mice, TG level of eWAT also exhibited significantly higher while free fatty acid (FFA) was lower when ColXV overexpressed (Supplementary Figure 2D and 2E). In addition, the increased mRNA level of adipogenic markers C/EBPβ, PPARγ and FABP4 by HFD were also strengthened in ColXV overexpressed group (Supplementary Figure 2F). Adipose tissue lipid metabolism related enzymes, such as fatty acid synthase (FASN) and acetyl coenzyme acarboxylase (ACCα) protein expressions were higher in Ad-Col15α1 group, while the phosphorylation of adipose triglyceride lipase (ATGL) and hormone sensitive lipase (HSL) were lower (Figure 2E). ELISA measurement of FASN, ACCα, ATGL and HSL showed the consistent results (Supplementary Figure 2G). Above results indicate ColXV is a positive regulator in mouse fat deposition, which could both accelerate lipid synthesis and weaken lipolysis *in vivo*.

### ColXV promoted cell differentiation in mice adipocytes

**Figure 3:**
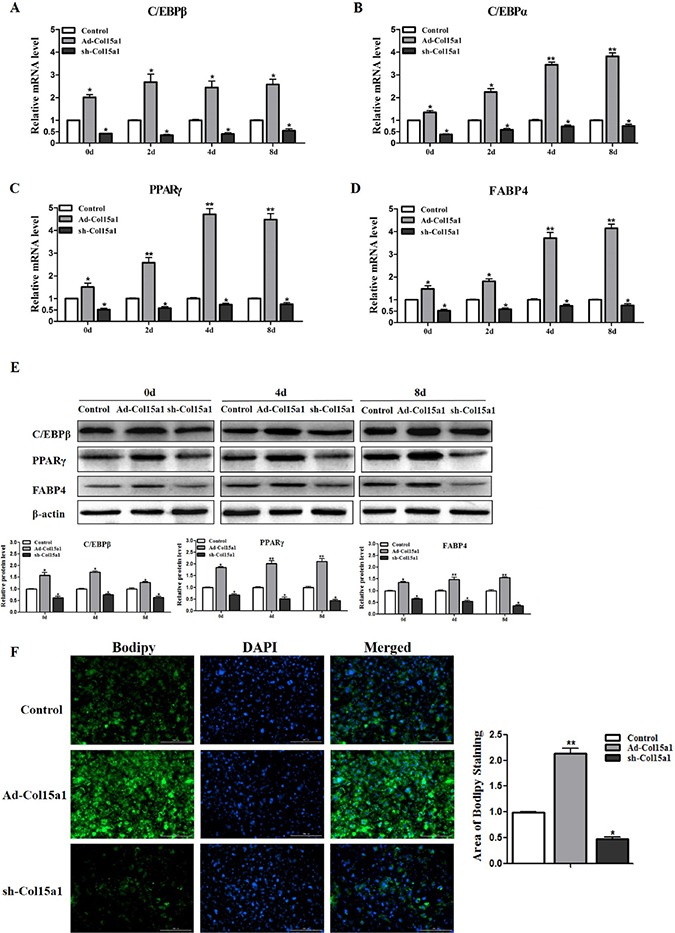
ColXV promoted cell differentiation in mice adipocytes Mice pre-adipocytes were infected with adenovirus over-expressed ColXV (Ad-Col15α1), adenovirus interfered ColXV (sh-Col15α1) or control (Ad-GFP) and then induced into mature adipocytes. On day 0, day 2, day 4 and day 8 of differentiation, mRNA levels of C/EBPβ (**A**) C/EBPα (**B**) PPARγ (**C**) and FABP4 (**D**) were measured respectively (*n* = 6). (**E**) C/EBPβ, PPARγ and FABP4 protein levels were detected on day 0, day 4 and day 8 of differentiation and quantified according to band density (*n* = 6). (**F**) Bodipy staining and quantitative analysis of lipid droplets in Ad-Col15α1, sh-Col15α1 and control infected adipocytes (*n* = 6). Data represent the mean ± SEM of triplicate. ^* #^*P* < 0.05, ^**##^*P*< 0.01.

We next addressed whether ColXV elicits accelerated effects on adipocyte differentiation and lipid synthesis *in vitro*. ColXV overexpression vector Ad-Col15α1 and interference vector sh-Col15α1 were used to control ColXV expression, Ad-GFP as control. There were no difference between Ad-GFP and negative control on ColXV expression, and Ad-Col15α1 and sh-Col15α1 were functional (Supplementary Figure 3A and 3B). We found overexpressing ColXV increased both mRNA levels (Figure 3A–3D) and protein levels (Figure 3E) of C/EBPβ, C/EBPα, PPARγ and FABP4 after 0 d, 4 d and 8 d differentiation and ColXV promoted adipocyte differentiation more remarkable in middle and later period. Greater amount of lipid droplets of adipocytes in ColXV overexpressed group relative to control were seen as determined by Bodipy staining, which was opposite to ColXV interference group (Figure 3F). These results mean ColXV promoted mice adipocyte differentiation mostly in middle and later stage.

### ColXV weakened isoproterenol-induced lipolysis in mice adipocytes

**Figure 4:**
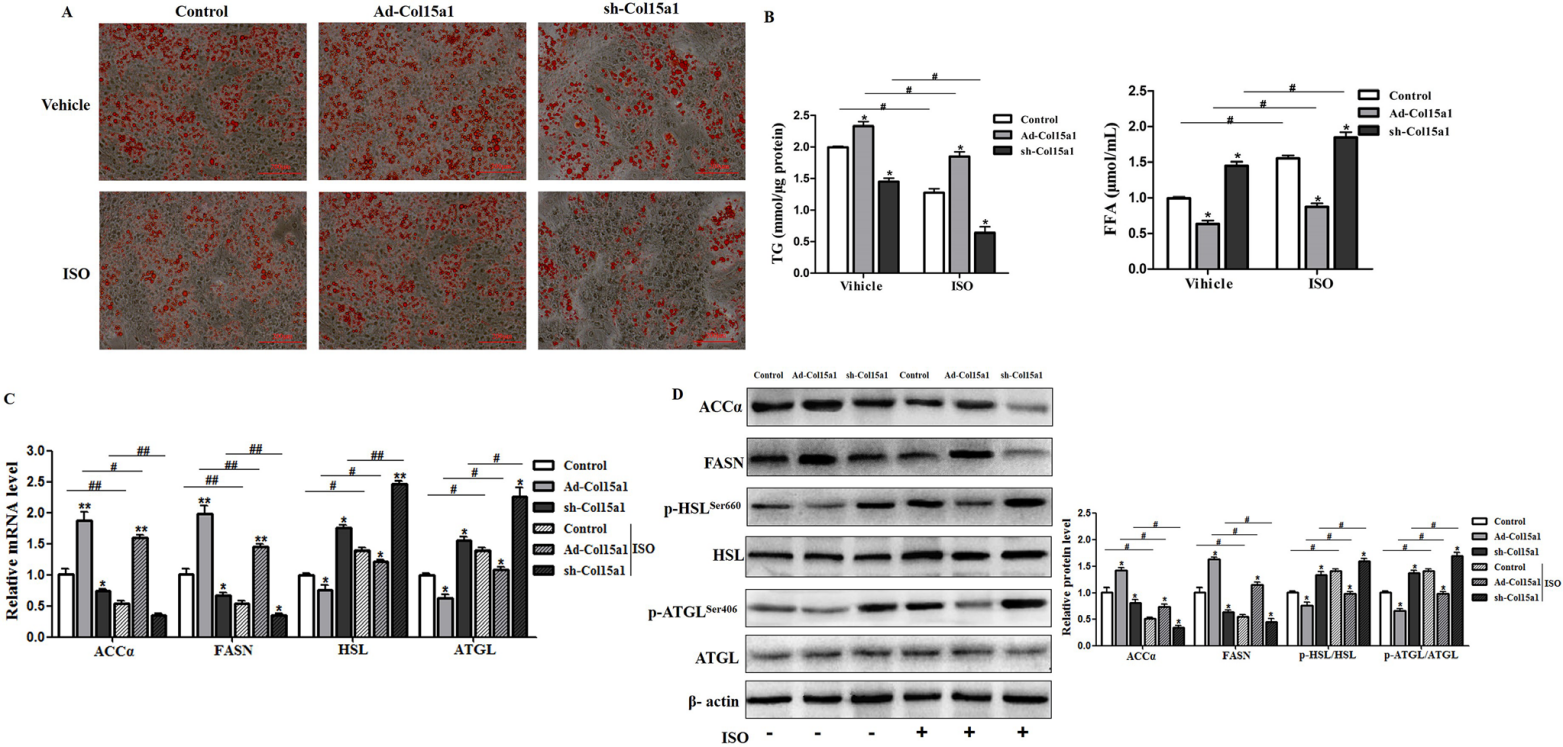
ColXV weakened isoproterenol-induced lipolysis in mice adipocytes Mice adipocytes were infected with Ad-Col15α1, sh-Col15α1 or control. Oil Red O staining (**A**) were carried out in mature adipocytes with respective treatment mentioned above (*n* = 6). Base on infections with different adenovirus, cells were treated with ISO or its vehicle DMSO. Then, TG and FFA concentration (**B**) relative mRNA levels of ACCα, FASN, ATGL, HSL (**C**) relative protein level of ACCα, FASN (**D**) and ATGL, HSL phosphorylation levels were all detected and quantified (*n* = 6). Data represent the mean ± SEM of three repeats. ^*#^*P* < 0.05, ^**##^*P* < 0.01.

To better understand the role of ColXV in adipocyte metabolism, we measured the expressions of lipogenesis and lipolysis genes in later differentiated stage. Overexpressed ColXV accelerated both the mRNA levels (Supplementary Figure 3C) and protein levels (Supplementary Figure 3D) of lipid synthesis genes, such as FASN and ACCα, while declined both mRNA level and phosphorylation of lipolysis genes, like HSL and ATGL. Isoprenaline (ISO), an adrenergic receptor agonist, has been used to induce lipolysis [22]. We treated adipocytes with ISO, intend to investigate the function of ColXV in ISO-induced lipolysis. Ablated effects were found with ISO treatment in ColXV over-expressed adipocytes, such as enhanced lipid droplet formation (Figure 4A), elevated dissociative TG level and decreased dissociative FFA level (Figure 4B). Overexpression of ColXV also blunted the lipolysis effect of ISO by rescuing the decreased FASN and ACCα level and repressing the increase of HSL and ATGL from both mRNA (Figure 4C) and protein phosphorylation level (Figure 4D). Our findings are consistent with previous studies that HSL and ATGL are phosphorylated in ISO-induced lipolysis process, at serine 660 for HSL [23] and at serine 406 for ATGL [22]. Our data revealed that ColXV enhanced adipocyte lipid synthesis and weakened ISO-induced lipolytic activity.

### CREB attenuated ColXV transcription in adipocyte differentiation and lipolysis

**Figure 5:**
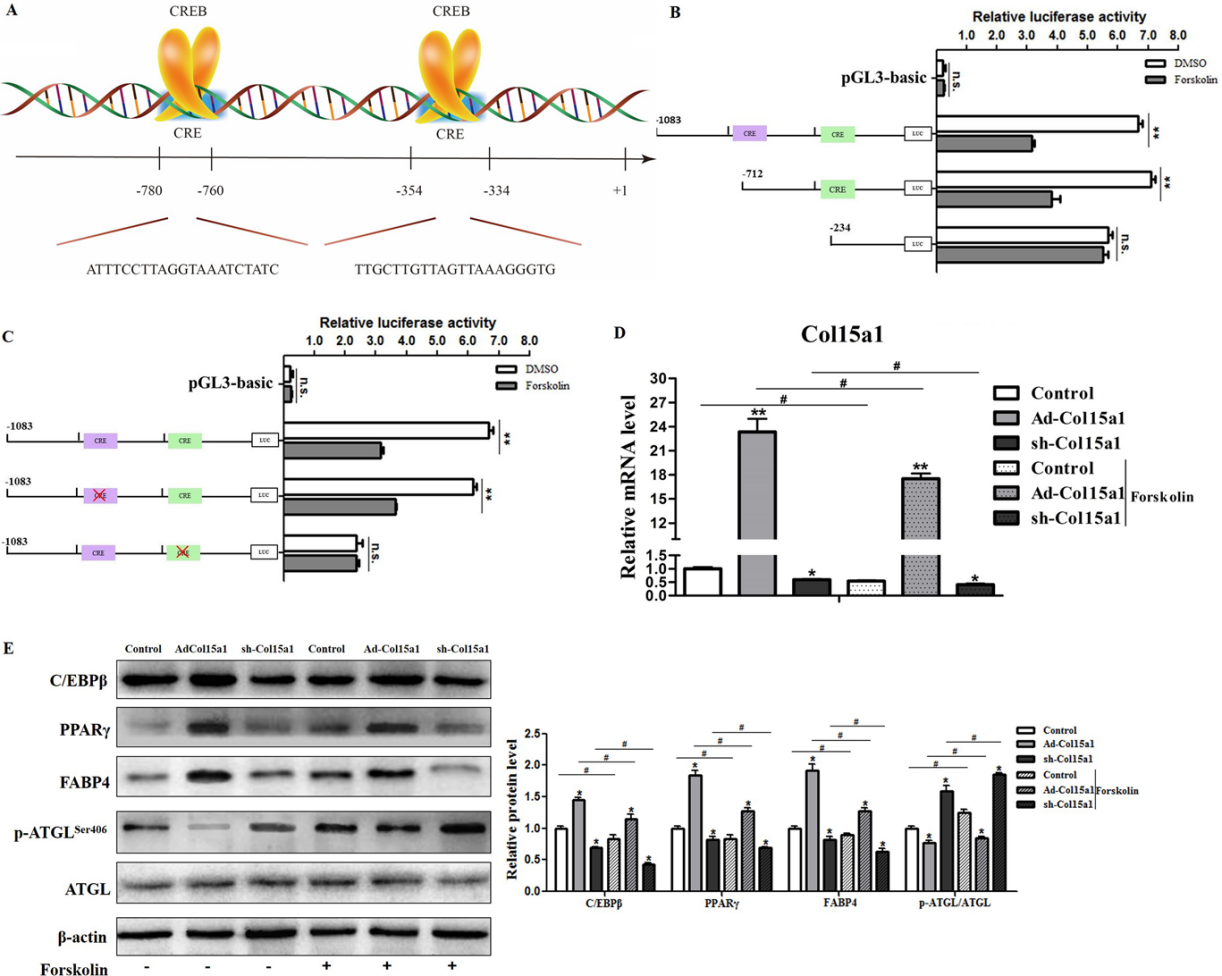
CREB attenuated ColXV transcription in adipocyte differentiation and lipolysis CREB was selected as candidate transcription regulator for ColXV according to analyzing results in Genomatix software. (**A**) The schematic diagram of CREB binding sites on ColXV promoter. Luciferase reporter gene fused ColXV promoter fragments (**B**) or mutated the CREB binding sites on ColXV promoter fragments (**C**) were transfected into cells with or without Forskolin treatment (*n* = 6). Luciferase activity was detected and corrected with Renilla luciferase activity (*n* = 6). To explore the function of CREB on ColXV expression, Forskolin was added in cells overexpressed or interfered ColXV, then relative ColXV mRNA level (**D**) were measured, together with protein levels of adipogenic genes, such as C/EBPβ, PPARγ, FABP4 and ATGL (**E**) were detected (*n* = 6). Data represent the mean ± SEM of triplicate. ^*#^*P* < 0.05, ^**##^*P* < 0.01.

In order to analyze the underlying mechanisms of ColXV on adipocyte differentiation and metabolism, we determined the transcription-level regulation. With Genomatix software analysis, CREB was found to be one of transcription factors of ColXV promoter (Supplementary Figure 4A), on which there were two potential cAMP response elements (CREs) for CREB binding, −354 bp ∼ −334 bp and −780 bp ∼ −760 bp upstream of transcription start site on ColXV promoter (Figure 5A). Then we carried out dual-luciferase reporter assay to verify the result of software prediction. For plasmids carrying the two binding regions and only −354 bp ∼ −334 bp region, when CREB was induced by forskolin, luciferase activity was lower than DMSO control (Figure 5B), which means both of the two regions could be functional and CREB has a negative role in regulating ColXV promoter activity. Further we used site-directed mutation to specify which binding site is critical for CREB regulation. When site −780 bp ∼ −760 bp was mutated, the blocking effect of ColXV transcription was still exist by Forskolin treatment, but when mutated the other binding site −354 bp ∼ −334 bp the blocking effect disappeared (Figure 5C). This implicated that the region −354 bp ∼ −334 bp is the functional binding site of CREB. Thus we suppose CREB may repress ColXV transcription and block its promoting function in adipocyte differentiation and metabolism. Adipocytes were overexpressed or interfered with ColXV and treated with Forskolin. Data showed CREB was successfully induced with Forskolin treatment (Supplementary Figure 4B) and CREB elevation blocked the transcription of ColXV (Figure 5D). Then, the elevated protein expressions of C/EBPβ, PPARγ, FABP4, and decreased phosphorylation level of ATGL caused by ColXV overexpression were greatly repressed by Forskolin treatment (Figure 5E). The mRNA expressions of C/EBPβ, PPARγ, FABP4 and ATGL presented the same trend (Supplementary Figure 4C). Thus, our findings showed that ColXV increased adipocyte differentiation and inhibited lipolysis through CREB negative transcription.

### ColXV enhanced adipocyte differentiation and weakened lipolysis via reducing DNA methylation

**Figure 6:**
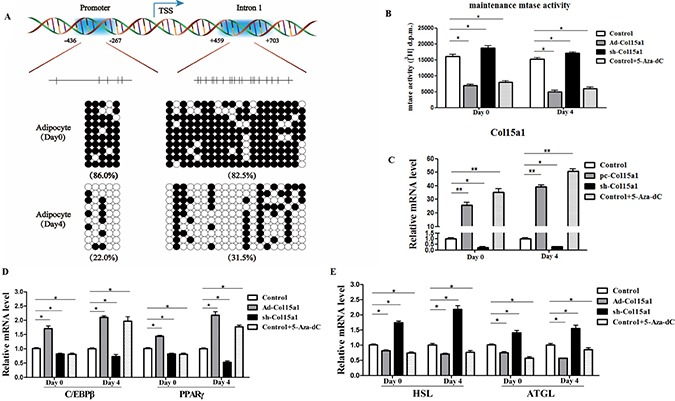
ColXV enhanced adipocyte differentiation and weakened lipolysis via reducing DNA methylation (**A**) Bisulphite sequencing analyses of the ColXV DNA. The methylation sites on promoter and intron 1 of ColXV are depicted. The CpGs in each region are represented with vertical bars. Methylation status of adipocytes was analyzed before (day 0) and after (day 4) differentiation induction (*n* = 3). Methylation status of the CpGs within each amplified regions were represented with closed circle (methylated) and open circle (unmethylated). (**B**) Maintenance methyltransferase (mtase) Dnmt1 activity was detected in control, Ad-Col15α1, sh-Col15α1 or 5-Aza-dC treated adipocytes, either on day 0 or day 4 after differentiation (*n* = 3). 5-Aza-dC or overexpressed and interfered with ColXV treated adipocytes, then relative Col15α1 mRNA level (**C**) were measured. Relative mRNA levels of C/EBPβ, PPARγ (**D**) and HSL, ATGL (**E**) were also detected. Data represent the mean ± SEM of three independent experiments. ^*#^*P* < 0.05, ^**##^*P* < 0.01.

DNA methylation downregulates gene transcription by controlling transcription start sites regulated by methyltransferase 1 (Dnmt1) [24]. Thus we studied DNA methylation of ColXV in adipocyte differentiation, also intended to clarify the relationship between ColXV and Dnmt1. Two methylation regions were predicted in ColXV DNA sequence with Laboratory of Molecular Medicine website, 5 CpGs present in ColXV promoter and 20 CpGs in the intron 1, respectively (Figure 6A). In this study, we found most methylation sites both in promoter and intron 1 of ColXV in differentiated mature adipocytes were lowly methylated compared with that in un-differentiated adipocytes (Figure 6A, Supplementary Figure 5A and 5B). This is consistent with our previous finding that ColXV expression was increased during adipocyte differentiation. To further investigate how DNA methylation function on ColXV regulated adipocyte differentiation and lipolysis, we treated adipocytes with 5-aza-2′-deoxycytidine (5-Aza-dC), a pharmacological inhibitor of Dnmt1 to induce demethylation [24]. Maintain methyltransferase Dnmt1 activity was inhibited by 5-Aza-dC both in pre-adipocytes and differentiated adipocytes, and ColXV overexpression group had a similar effect (Figure 6B). However, de novo methyltransferases Dnmt3a and Dnmt3b activity were unaffected in ColXV or 5-Aza-dC treatment (Supplementary Figure 5C). Adding of 5-Aza-dC reduced the methylation levels in both promoter and intron 1 of ColXV (Supplementary Figure 5D and 5E), which correspondingly elevated ColXV mRNA level (Figure 6C). Then we analyzed the expressions of adipogenic and lipolytic markers after infecting adipocytes with Ad-Col15α1/sh-Col15α1 or treating with 5-Aza-dC. In early differentiation period, ColXV promoted C/EBPβ and PPARγ mRNA expressions, while 5-Aza-dC inhibited them (Figure 6D), which is opposite with later stage differentiation. This can be explained by the inhibitory effect of 5-Aza-dC on adipogenesis in early differentiation stage [25]. Lipolytic markers, such as HSL and ATGL mRNA expressions were all blunted after either ColXV overexpression or adding 5-Aza-dC, regardless of early or later differentiation period (Figure 6E). Similarly, enhanced lipid droplet formation (Supplementary Figure 5F), elevated TG level and decreased FFA level (Supplementary Figure 5G) were observed in 5-Aza-dC, also similar to ColXV forced expression group. Our results suggested that ColXV has a similar effect with 5-Aza-dC on DNA demethylation and this can be one mechanism in ColXV induced adipocyte differentiation and weakened lipolysis.

### cAMP/PKA signal pathway was essential for effects of ColXV on adipocyte differentiation and lipolysis

**Figure 7:**
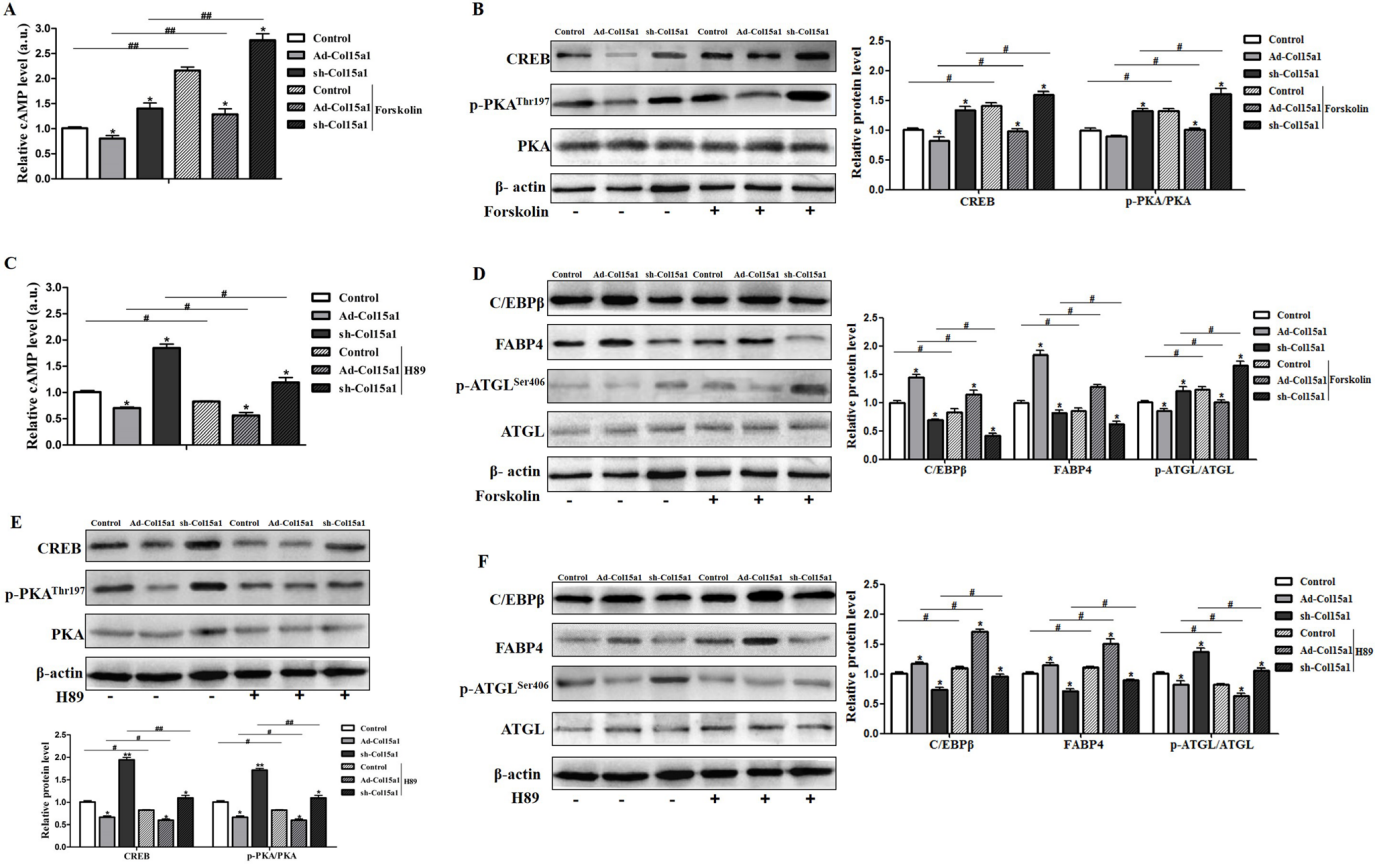
cAMP/PKA signal pathway was essential for effects of ColXV on adipocyte differentiation and lipolysis Adipocytes were pre-infected with Ad-Col15α1 and sh-Col15α1 (Ad-GFP as control) for 72 h. Part of those cells were incubated with 10 μM Forskolin for 1 h, then cAMP level (**A**) protein level of CREB, p-PKA/PKA (**B**) and C/EBPβ, FABP4, ATGL, p-ATGL^Ser406^ (**D**) were detected (*n* = 6). Another part of those cells were treated with 10 μM H89 for 2 h, followed by cAMP level (**C**) protein level of CREB, p-PKA and PKA (**E**) and C/EBPβ, FABP4, ATGL, p-ATGL^Ser406^ (**F**) evaluation (*n* = 6). Data represent the mean ± SEM of three independent experiments. ^*#^*P* < 0.05, ^**##^*P* < 0.01.

To clear the molecular mechanism underling the regulation of ColXV and CREB on adipocyte differentiation, we studied the function of cAMP/PKA signal pathway in CREB-ColXV mediated adipocyte metabolism using pathway activator and inhibitor. When cAMP/PKA signal pathway was activated by Forskolin, key factors in cAMP/PKA signal pathway such as cAMP (Figure 7A), CREB and p-PKA (Figure 7B) were correspondingly increased, while pathway inhibitor H89 brought the opposite effects (Figure 7C and 7E), which means the cAMP/PKA signal pathway was successfully activated or inhibited.

We found ColXV overexpression promoted adipocyte differentiation by elevating adipogenic markers C/EBPβ and FABP4 protein levels, reducing lipolytic marker ATGL phosphorylation. While adding of Forskolin could negatively regulate the effects caused by ColXV and the anti-differentiation effect of ColXV interference was found enhanced by Forskolin (Figure 7D). When cAMP/PKA signaling pathway is inhibited by H89, the differentiation inducement effect of ColXV overexpression was further enhanced and the anti-differentiation effect of ColXV interference was weakened (Figure 7F). Hence, these data indicated the effect of ColXV in increasing adipocyte differentiation and decreasing lipolysis was by inhibiting cAMP/PKA signal pathway.

## DISCUSSION

ECM is an important multifunctional molecular content, which provides structural support to tissues and organs, modifies inter/extracellular signals, and regulates essential cellular functions [7–10]. Studies indicated that excessive accumulation or inappropriate remodeling of ECM in adipose tissues may lead to obesity or even adipose dysfunction [26–28]. ECM components especially collagens play an important role in adipose metabolism. Collagens turnover in pre-adipocyte differentiation through metalloproteinases (MMPs) and their tissue inhibitors (TIMPs) regulate adipose tissue remodeling and whole-body lipid distribution [29]. Different collagen components were secreted in various stages of adipocyte differentiation [30]. In this study, we demonstrated that ColXV expression was elevated in mature adipocytes and adipose tissues of HFD fed mice, the higher expression could further accelerate lipid deposition in obese mice. This is consistent with previous report that proteome ColXV is up-regulated during adipogenesis in a temporal fashion [31]. ColXV and ColXVIII are considered as MULTIPLEXINs/endostatin-producing collagens. Previous studies have shown ColXVIII directly regulate adipocyte differentiation, fat deposition and lipolysis [16–18]. Here, we found ColXV promoted adipocytes differentiation mostly in middle and later stage and in the meantime, enhanced adipocyte lipid synthesis and weakened ISO-induced lipolysis.

CREB is a well-documented transcription factor involved in adipocyte differentiation and lipid metabolism. Previous study has shown collagen triple helix repeat containing 1 (Cthrc1) inhibits adipocyte differentiation by inhibiting PPARγ signaling and activating CREB [32], which is consistent with our findings CREB as a negative regulator in ColXV promoted adipocyte differentiation. Although some reports indicated that CREB participates in adipogenesis via positive transcriptional regulating C/EBPβ and PPARγ at early state [33, 34], we found CREB suppressed adipocyte differentiation dependent on ColXV. Moreover, CREB was found binding on −354 bp ∼ −334 bp upstream of ColXV promoter, thus attenuate ColXV transcription and reverse the effects of ColXV on adipocyte differentiation and lipolysis.

An interesting observation from bisulfite sequencing was that lower methylation level presented both in promoter and intron 1 of ColXV after adipocytes differentiation. Past studies indicated that DNA methyltransferases are important in maintaining methylation patterns during adipocyte differentiation, altering fat tissue remodeling and ameliorating obesity-induced glucose intolerance and insulin resistance [35–37]. In our study, we found ColXV has a similar effect with 5-Aza-dC on decreasing Dnmt1 activity during adipocyte differentiation. Interestingly, we demonstrated ColXV had no effects on de novo methyltransferase activity, which is consistent with previous reports that inhibition of Dnmt1 but not Dnmt3a or Dnmt3b function by 5-aza-2-deoxycytidine is the principal means to reactivate genes [38]. Dnmt1, as an enzyme that responsible for maintaining DNA methylation, forms part of the family of DNA methyltransferase enzymes. The reduction of Dnmt1 expression could lead to a low methylation status for genes, including adipogenic and lipolytic genes, thus induce their expressions. So, we continued to investigate whether the inhibition of Dnmt1 activity by ColXV further influence adipocyte differentiation. Data show that adipocyte differentiation was promoted and lipolysis was decreased when ColXV was over-expressed and this effect was accordant with 5-Aza-dC. Our data suggested the down-regulation of Dnmt1 by ColXV could be one reason in its role in promoting adipocyte differentiation and inhibiting lipolysis.

The cAMP/PKA signal pathway has been reported to be involved in adipocyte lipolysis activation and adipocyte differentiation inhibition [39–41]. CREB is also activated by PKA-mediated phosphorylation and activation of PKA and CREB leads to the phosphorylation of HSL^Ser660^ and ATGL^Ser406^, which facilitates lipolysis process [22, 23]. Because of the repressing effect of CREB on ColXV, we detected the role of cAMP/PKA signal pathway in ColXV function. Results show that the promoting effect of ColXV on adipogenesis and repressing effect on lipolysis is through inhibiting cAMP/PKA signaling pathway, consistent our previous finding that cAMP/PKA signal pathway activation inhibited adipogenic markers C/EBPβ, PPARγ and FABP4 [42].

**Figure 8:**
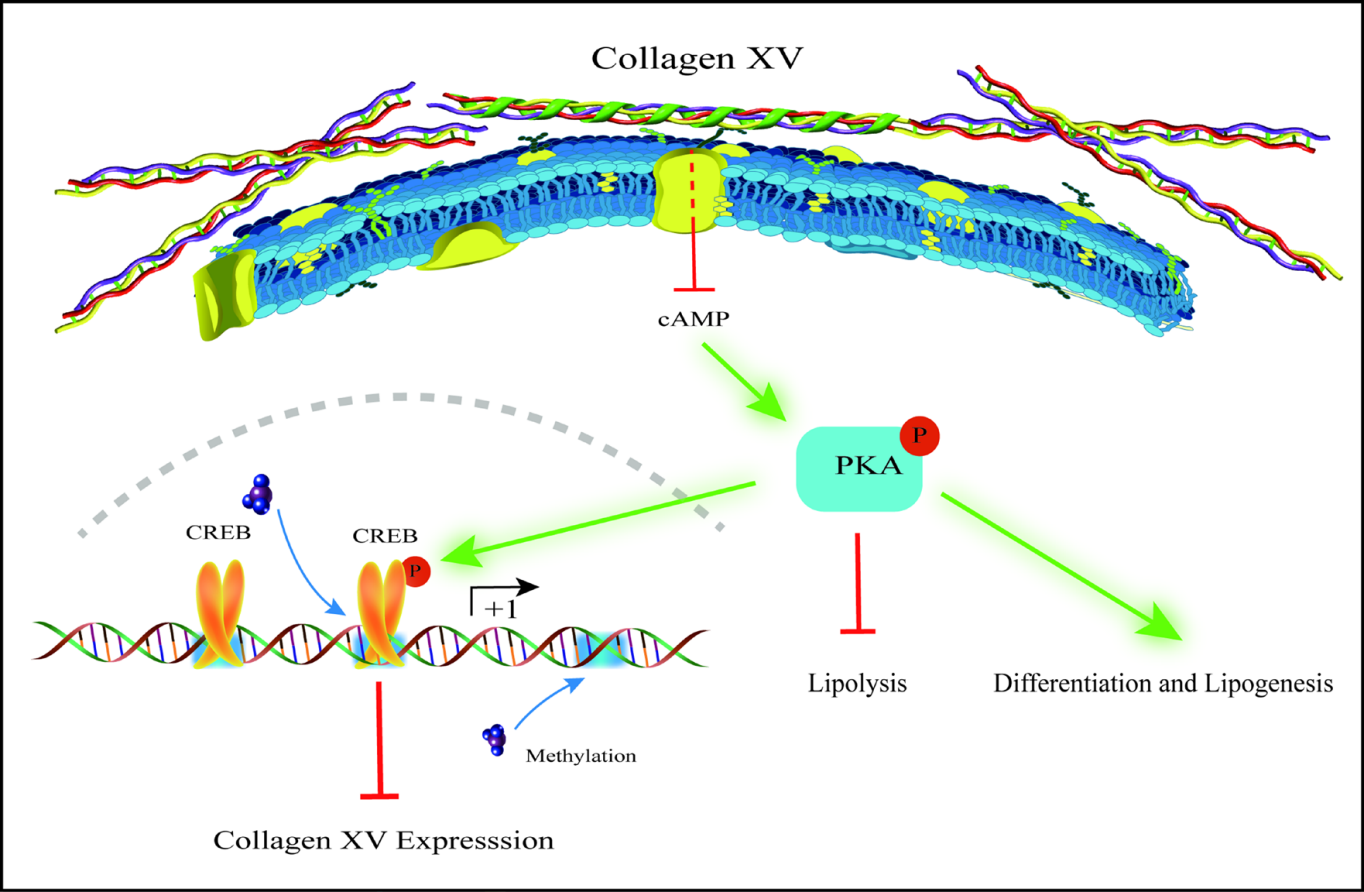
Proposed model for mechanism of ColXV on adipocyte differentiation and lipolysis ColXV expressions were increased in adipose tissues of obese mice and mature adipocytes. ColXV aggravated mice obesity. *In vitro*, ColXV promoted adipocyte differentiation and decreased adipocyte lipolysis. ColXV promoted adipocyte differentiation and enhanced lipolysis via inactivating the phosphorylation of cAMP/PKA signal pathway, and CREB reversed the effects of ColXV on adipocyte differentiation via binding its promoter. In addition, ColXV promoted adipocyte differentiation though inhibiting DNA methylation. Above all, ColXV accelerated adipocyte differentiation and inhibited lipolysis.

In summary, our findings provide compelling evidence that ColXV promoted adipocyte differentiation and inhibited lipolysis via cAMP/PKA pathway. Moreover, we found that CREB was a novel transcriptional suppressor of ColXV and reduce ColXV function of promoting adipocyte differentiation and alleviating lipolysis. ColXV could promote fat deposition and weaken lipolysis by inhibiting its DNA methylation (Figure 8). Our results might contribute to further understanding of regulatory mechanisms of adipocytes differentiation and lipolysis to seek for novel approaches to overcome obesity.

## MATERIALS AND METHODS

### Animal experiments

Two-week-old male Kunming mice were purchased from the Laboratory Animal Center of the Fourth Military Medical University. All mice were treated in accordance with the applicable guidelines and regulations approved by the Animal Ethics Committee of Northwest A&F University. Mice were allowed to ad libitum water and standard chow diet (Control, fat provides 10% of total energy) or high fat diet (HFD, fat provides 60% of total energy) for 10 weeks. Body weight and food intake were recorded each week. The animal room was maintained in controlled conditions of temperature at 25 ± 1°C, humidity at 55 ± 5%, and a 12 h light and 12 h dark cycle. After intraperitoneal injection of recombinant adenovirus overexpression vector of ColXV (Ad-Col15α1) and recombinant adenovirus interference vector of ColXV (sh-Col15α1) to HFD or chow diet fed mice for 10 days, mice were euthanized for collection of tissues and blood. eWAT, iWAT and BAT were sampled in epididymis, inguinal and interscapular adipose tissue, respectively.

### Body composition analysis

Mouse body composition analysis of HFD or chow diet fed mice at 9 weeks old was performed using a small animal MRI (Echo Medical Systems, Houston, USA). Fat and lean depots were quantified (*n* = 7∼8 per group).

### Cell culture

Mouse primary adipocytes were harvested from epididymal white adipose tissues of four-week-old mice, and HEK293A and HEK293T cells were saved in our lab. Cells was maintained in proliferation medium (PM) containing Dulbecco's modified Eagle's-H21 medium/Ham's F12 (DMEM/F12), 10% FBS (Gibco, Carlsbad, USA) and 1% penicillin-streptomycin in a humidified atmosphere of 5% CO_2_ at 37°C. To induce pre-adipocytes into mature adipocytes, cells were grown in differentiation medium (DM) containing 1 μM dexamethasone (Sigma, MO, USA), 0.01 mg/mL insulin (Sigma, MO, USA), 0.5 mM isobutylmethyl xanthine (Sigma, MO, USA), 10% FBS (Gibco, Carlsbad, USA) and 1% penicillin-streptomycin 2 days after confluence. After 2 days differentiation, cells were then maintained in DMEM/F12, 10% FBS, 1% penicillin-streptomycin, and 0.01 mg/mL insulin. Cells were differentiated after induction for 6 days.

### Chemical treatment and infection

The recombinant adenovirus vector Ad-Col15α1 and adenovirus interference vector sh-Col15α1 were infected for 24 h or 48 h at the titer of 1 × 10^9^ IFU/mL to test the effect of the changes of ColXV expression on adipocyte differentiation. Gene expressions of adipocyte differentiation and lipolysis makers were detected by Quantitative Real-Time PCR or Western blot. When testing the impact of ColXV on cAMP/PKA signal pathway activated by Forskolin (10 μM, Selleck, Houston, USA), differentiated adipocytes were treated with H89 (10 μM, Selleck, Houston, USA) or DMSO (Promega, Madison, USA) 1 h prior to Forskolin (10 μM), and samples were assessed by Western blot. Experimental procedure was as described in our previous reports in detail [42].

### Bodipy staining

Bodipy (Invitrogen, Carlsbad, USA) with 513 nm green florescent was diluted with DMSO to a concentration of 1 mm as work solution. When differentiating for 6 d, adipocyte lipid droplets were stained with Bodipy. In brief, cells were rinsed three times with PBS and then fixed in 10% (vol/vol) formaldehyde for 30 min. The fixed cells were then washed three times in PBS and stained with a working solution of Bodipy for 30 min at room temperature. Cells were washed with PBS thrice and image observation was taken with an inverted microscope (Nikon Instruments Europe BV, England).

### Oil red O staining

Lipid droplets were stained with Oil Red O staining as described in our previous publication [43]. Firstly, primary adipocytes were washed with PBS thrice, and then incubated in 4% formaldehyde for 30 min at room temperature. Then cells were gently washed with PBS thrice, stained with Oil Red O (Sigma, St. Louis, USA) for 30 min, and incubated at 37°C temperature. Cells were washed in PBS for 3 times to remove unbounded dye, and then took photographs with Nikon TE2000-S florescent microscope.

### TG and FFA measurement

Total protein content was measured with the DC assay (BioRad, USA) and triglycerides (TGs) in adipose tissue or adipocytes were analyzed by TG assay kit (Jiancheng, Nanjing, China). Free fatty acid (FFA) content was determined at absorption at 570 nm with FFA assay kit (Jiancheng, Nanjing, China). Determination methods of TG and FFA were consistent with our previous studies [44].

### Enzyme-linked immunosorbent (ELISA) assay

Adipocytes and adipose tissue were collected as our study described previously [45]. Briefly, cells after Ad-Col15α1 and sh-Col15α1 infection for 96 h were collected and disrupted by ultrasonication (28 KHz, 30 min). After induced differentiation for 4 days cAMP level in adipocytes and the protein level of FASN, ACCα, ATGL and HSL in white adipose tissue were measured using commercial ELISA kits (R&D Systems, USA).

### Lipolysis experiments *in vitro*

Primary cultured adipocytes were serum-starved in Krebs–Ringer buffer containing HEPES (KRH buffer) (NaCl at 120 mM; KCl at 4.7 mM; CaCl_2_ at 2.2 mM; HEPES at 10 mM; KH_2_PO_4_ at 1.2 mM; MgSO_4_ at 1.2 mM; glucose at 5.4 mM) supplemented with 1% bovine serum albumin (BSA) fatty acid free (Roche, Switzerland) for 3 h. Following starvation, the cells were incubated with KRH buffer containing 4% BSA in the presence and absence of isoproterenol (Sigma, USA) at concentrations of 1 μM. All adipocytes lipolysis measurements were made over a 3 h period at 37°C and 5% CO_2_.

### Promoter reporter assay and dual luciferase reporter assay

Three fragments containing ColXV - 5′ sequences −1083 bp ∼ +40 bp, −712 bp ∼ +40 bp and −234 bp ∼ +40 bp relative to the transcription initiation site were sub-cloned into pGL3-basic vector, respectively (Takara, China). Mutant ColXV reporter plasmids were generated using the luciferase plasmid contained −1083 bp ∼ +40 bp fragment as a template, then a mutagenesis kit (Invitrogen, CA, USA) was used to created site-directed mutagenesis for two CREs, as described in our previous study [46]. HEK293T cells were cultured in 24-well plates and co-transfected with ColXV reporter plasmid and pRL-TK plasmid (control reporter), then treated with Forskolin or DMSO for 6 h. Cells were harvested 48 h after transfection, and detected using the Dual-Luciferase Reporter assay system (Promega, USA). Dual luciferase reporter assay were as previously described [47].

### Bisulfite conversion and sequencing

The methylation of ColXV was analyzed by bisulfite sequencing. Adipocyte and adipose tissue genomic DNA was extracted with Maxwell^®^ RSC Cultured Cells DNA Kit (Promega, USA). Three DNA pools from each group were performed by sodium bisulfite treatment using the EZ DNA Methylation Kit (Zymo Research, USA). The primers were designed by online Methprimer software (http://www.urogene.org/cgi-bin/methprimer/methprimer.cgi). The primers in ColXV promoter for bisulfite sequencing PCR (BSP) were as follows: forward TTTATTGGATGGATGTTTTGGTAA, reverse CCACTTACATACCCCAAAATAACAC. While the primers in ColXV intron 1 were as follows: forward GAGGTGGTTGTTTTTTATTATTTT, reverse AACCCAAACACTCTTATATACCTTC. Modified genomic DNA was served as the template for PCR amplification immediately and PCR products were gel purified using Gel Purification Kit (Omega, USA). Then purified DNA were cloned into the pMD19-T vector (Takara, China) and then transformed into Escherichia coli. Later, competent cells were plated on lysogeny broth (LB) solid medium and identified by blue-white selection. Positive clones for each subject were randomly selected for sequencing (Invitrogen, China).

### DNMT activity assay

Adipocytes were infected with recombinant adenovirus vector Ad-Col15α1 and interference vector sh-Col15α1. Another part of adipocytes were cultured in fresh complete culture medium with 10 μM 5-aza-dC as positive control. We detected DNMTs activity on nuclear extracts using 0.2 μg of a synthetic 33-bp hemi-methylated oligonucleotide containing 8 CGs and 0.55 mCi of S-[methyl-^3^H] adenosyl-L-methionine (75 Ci mmol^−1^) in a 20 mM Tris-HCl (pH 7.6), 10 mM EDTA, 25% glycerol and 1 mg mL^−1^ bovine serum albumin buffer. We stopped reactions using 12% trichloroacetic acid, transferred samples onto a filter mat, washed them with cold 10% trichloroacetic acid, dried them and counted ^3^H using a Wallac beta-counter.

### Quantitative real-time PCR analysis

Total RNA was extracted with TRIpure Reagent kit (Takara, China). 500 ng of total RNA was reverse transcribed using M-MLV reverse transcriptase kit (Takara, China). Primers were designed as Table 1 in Supplementary Data synthesized by Invitrogen (Shanghai, China). Quantitative PCR was performed in 20 μL reaction system containing specific primers, cDNA and SYBR Premix EX Taq (Takara, China). The levels of mRNA were normalized using β-actin. The expressions of genes were analyzed by the method of 2^−ΔΔCt^.

**Table 1:** Primers for quantitative real-time PCR

Genes	Accession number	Primer sequences (5′ to 3′)
C/EBPβ	NM_001287739.1	F: GCCCGTTGCCAGGCGCCGCCTTATAAA
		R: GGCTCCAGGTAGGGGCTGAAGTCGA
PPARγ	NM_001001460	F: CGAATGCCACAAGCGGAGAAGG
		R: CTTGGCTTTGGTCAGCGGGAA
C/EBPα	NM_001031459	F: ATGGAGCAAGCCAACTTCTAC
		R: GCCAGGAACTCGTCGTTGAA
FABP4	NM024406.2	F: AAGTGGGAGTGGGCTTTG
		R: GTCGTCTGCGGTGATTTC
FASN	NM_205155	F: AGTGTCCACCAACAAGCG
		R: GATGCCGTCAGGTTTCAG
ACCα	NM_133360.2	F: GGATATCGCATCACAATTGGC
		R: CCTCGGAGTGCCGTGCTCTGGATC
HSL	NM_001039507.2	F: AGACACCAGCCAACGGATAC
		R: GGGCATAGTAGGCCATAGCA
ATGL	NM_001163689.1	F: GACCTGATGACCACCCTTTC
		R: TGCTACCCGTCTGCTCTTT
UCP1	NM_009463.3	F: GCCAAAGTCCGCCTTCAGAT
		R: TGATTTGCCTCTGAATGCCC
Col15α1	NM_009928.3	F: TCCGAGATGGTTGGAAAAAG
		R: AAATGGGGTTCAGTGGAGGT
CREB	NM_133828.2	F: ACCCAGGGAGGAGCAATACAG
		R: TGGGGAGGACGCCATAACA
Dnmt1	NM_001314011.1	F: GGGTCTCGTTCAGAGCTG
		R: GCAGGAATTCATGCAGTAAG
GAPDH	NM_001289726.1	F: AGGTCGGTGTGAACGGATTTG
		R: TGTAGACCATGTAGTTGAGGTCA

### Immunoblot analysis

Adipocytes were solubilized in adipocyte lysing buffer. The solubilization was preceded for 40 min at 4°C, then the solution was centrifuged at 12,000 rpm for 15 min at 4°C, and the supernatants were used for determination of protein concentration. Protein samples (30 μg) were separated by electrophoresis on 12% and 5% SDS-PAGE gels using slab gel apparatus, and transferred to PVDF nitrocellulose membranes (Millipore, USA), blocked with 5% skim milk powder/Tween 20/TBST at room temperature for 2 h. The membranes were then incubated with primary antibodies against PPARγ (Ap0686), HSL (BS2742), ACCα (BS1378), β-actin (Ap0060) (Bioworld, CA, USA), C/EBPβ (ab32098), FABP4 (ab92501), ATGL (ab109251), CREB (ab7540), FASN (ab128856), PKA (ab108385), p-PKA^Thr197^ (ab75991), Col15α1 (ab150463), p-ATGL^Ser406^ (ab135093) (Abcam, Cambridge, UK), p-HSL^Ser660^ (4126) (Cell Signaling Technology, MA, USA) at 4°C overnight. Following this step, the appropriate HRP-conjugated secondary antibodies (Boaoshen, China) were added and incubated for 2 h at room temperature. Proteins were visualized using chemiluminescent peroxidase substrate (Millipore, USA), and then the blots were quantified using ChemiDoc XRS system (Bio-Rad, USA). Experimental procedure was as described previously [48].

### Statistical analysis

Statistical analyses were conducted by SAS v8.0 (SAS Institute, Cary, NC). Data were analyzed using either one-way ANOVA or two-way ANOVA depending on the number of variables. Comparisons among individual means were made by Fisher's least significant difference (LSD). Data were presented as mean ± SEM. *P* < 0.05 was considered to be significant.

## SUPPLEMENTARY MATERIALS FIGURES


