# Supplementary material for: ColXV promotes adipocyte differentiation via inhibiting DNA methylation and cAMP/PKA pathway in mice
Source: Oncotarget. 2017 Jun 16;8(36):60135–48. doi: 10.18632/oncotarget.18550 (PMC5601127; doi:10.18632/oncotarget.18550)
Supplement: Supplementary file 1 [file oncotarget-08-60135-s001.pdf]

ColXV promotes adipocyte differentiation via inhibiting DNA methylation and cAMP/PKA pathway in mice

Supplementary Materials

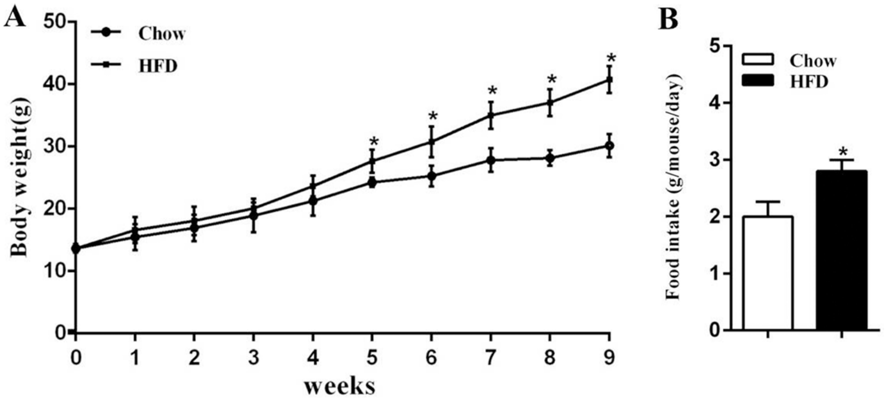

Supplementary Figure 1: The effect of HFD on body weight and food intake of chow and HFD fed mice. Mice were fed with HFD or chow diet for 9 weeks before collecting tissues. Body weight and food intake were recorded each week. Body weight (**A**) and average food intake (**B**) in HFD mice were compared with those in chow diet fed mice. $n = 6$. Data represent the mean ± SEM of three independent experiments. $^{*}P < 0.05$.

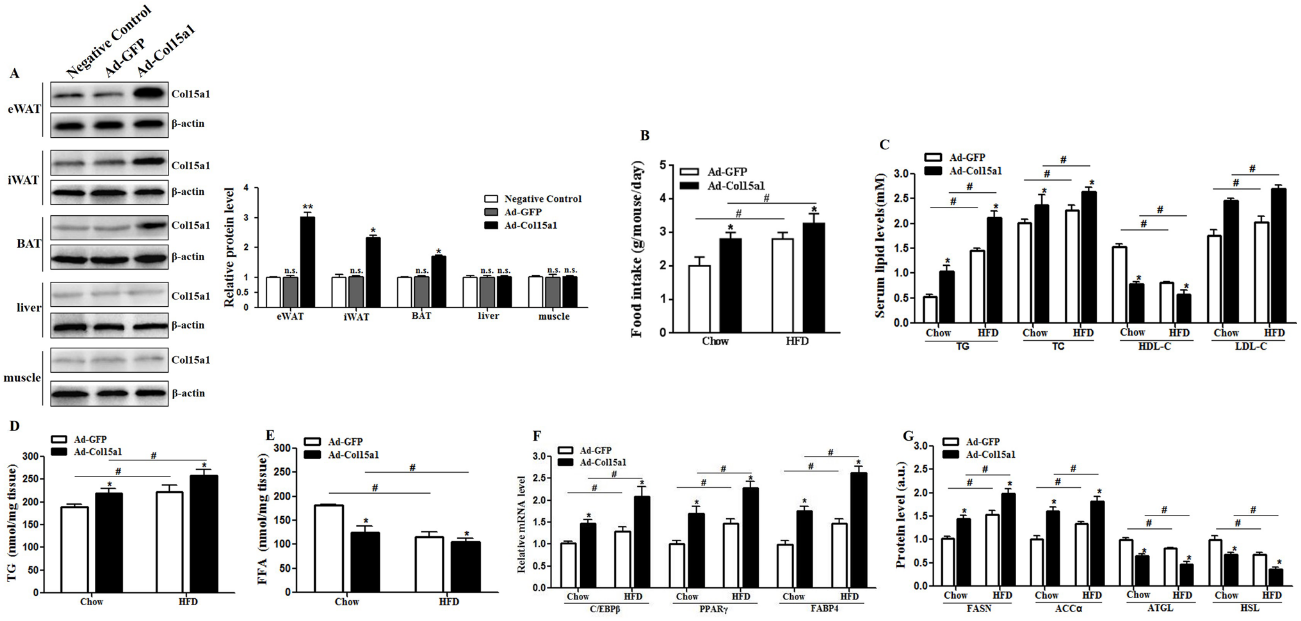

Supplementary Figure 2: The effect of ColXV on metabolic parameters, serum lipid, differentiation and lipid metabolism related genes level. Mice fed with chow and HFD diet were intraperitoneal injected with Ad-Col15α1 or Ad-GFP. ColXV protein expressions in eWAT, iWAT, BAT, liver and muscle (**A**) were detected after adenovirus injection compared with that in corresponding tissue of mice without adenovirus injection. Food intake (**B**) serum lipids (**C**) TG (**D**) and FFA (**E**) isolated from eWAT were detected. Relative mRNA levels of adipogenic markers (**F**) in mice eWAT fed on HFD and chow diet after Col XV overexpression were detected by Quantitative Real-Time PCR. Protein activity of lipid metabolism related genes in eWAT of HFD and chow diet fed mice after Col XV overexpression (**G**) were detected by ELISA. $n = 6$. Data represent the mean ± SEM of three independent experiments. $^{*\#}P < 0.05$, $^{**\#\#}P < 0.01$.

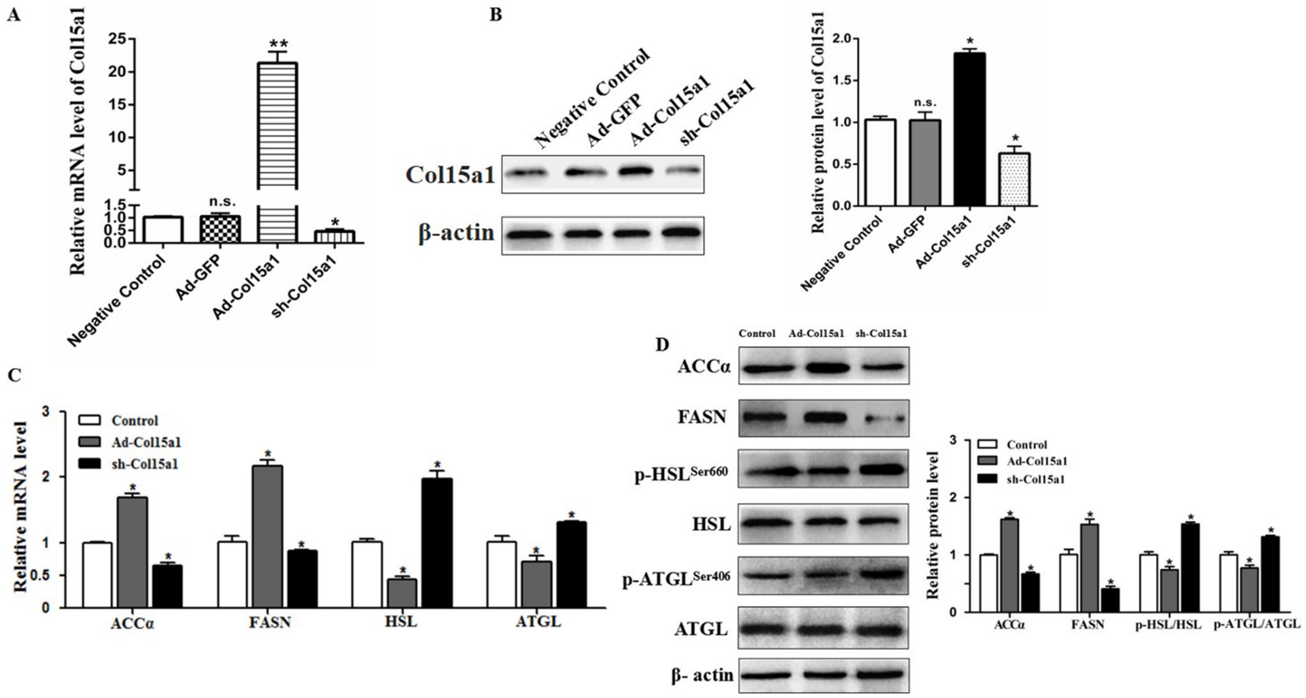

Supplementary Figure 3: ColXV overexpressed and interfered vector efficiency verification and effect of ColXV on lipolysis. Ad-Col15α1 and sh-Col15α1 or Ad-GFP infected adipocytes, and isolated RNA after 24 h or protein after 48 h for ColXV expression efficiency verification compared with negative control. Relative mRNA level (**A**) and protein level of ColXV (**B**) were determined. After infecting with Ad-Col15α1 and sh-Col15α1, relative mRNA level of FASN, ACCα, HSL and ATGL in mature adipocytes (**C**) and protein level (**D**) were measured, empty vector as control. $n = 6$. Data represent the mean ± SEM of three independent experiments. *$P < 0.05$.

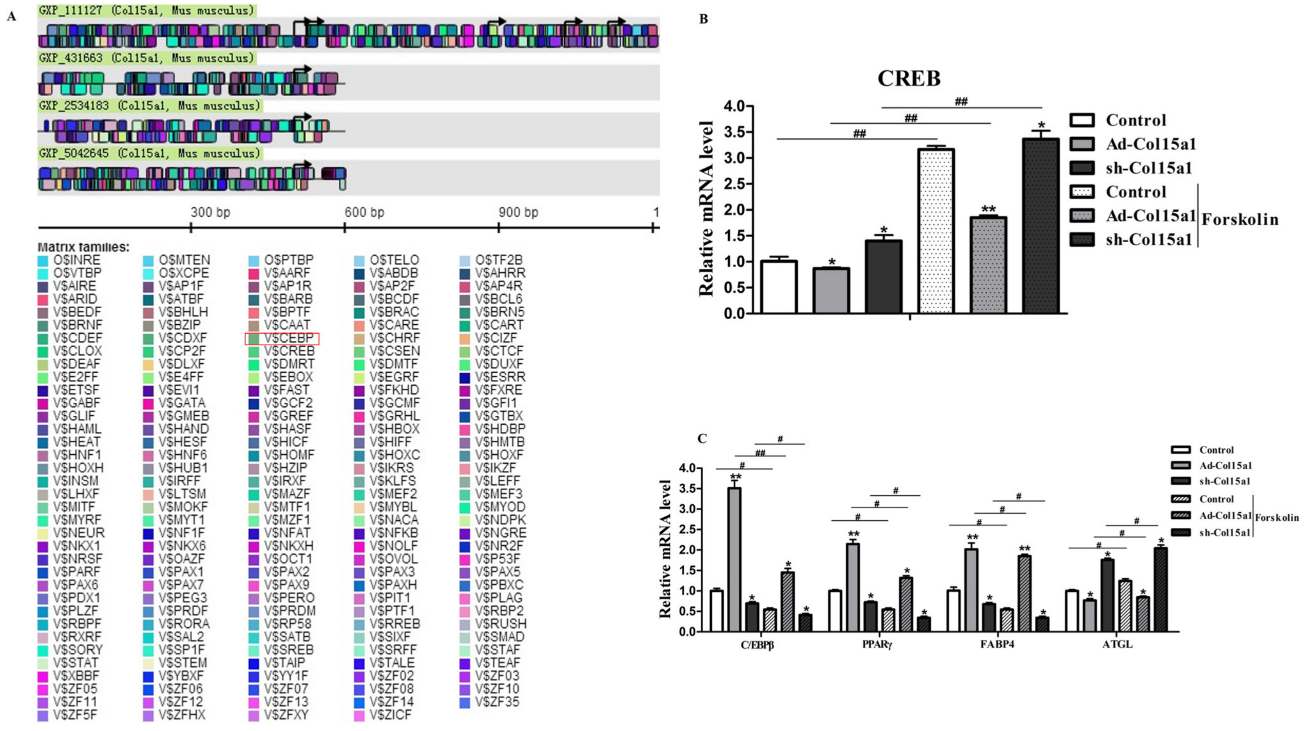

Supplementary Figure 4: Prediction for transcription factors on ColXV promoter and the effect of CREB agonist on adipogenic and lipolysis gene expressions. (**A**) Prediction for transcription factors on ColXV promoter with Genomatix software analysis, CREB in adipose tissue was one of them. After infecting with Ad-Col15α1 and sh-Col15α1, CREB agonist Forskolin were added to adipocytes for 12 h, CREB mRNA (**B**) and C/EBPβ, PPARγ, FABP4 and ATGL mRNA levels (**C**) were detected. $n = 6$. Data represent the mean ± SEM of three independent experiments. $^{*\#}P < 0.05$, $^{**\#\#}P < 0.01$.

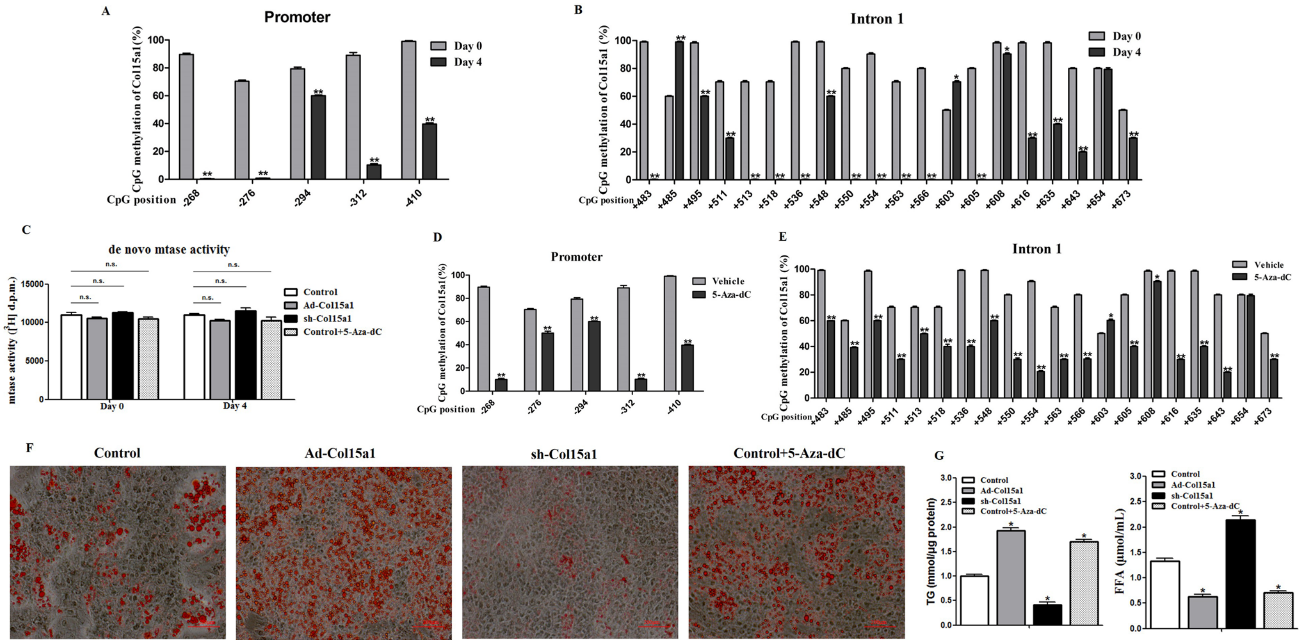

Supplementary Figure 5: The effect of ColXV on adipocyte differentiation compared with DNA methylation inhibition by 5-Aza-dC. Bisulphite sequencing analyses of the ColXV DNA. CpG methylation of ColXV promoter (**A**) and intron 1 (**B**) in adipocytes before or after differentiation (*n* = 3). (**C**) Adipocytes treated with 10 μM 5-Aza-dC or Ad-Col15α1 and sh-Col15α1, de novo methyltransferase (mtase) Dnmt3a and Dnmt3b activity was detected (*n* = 3). CpGs methylation of ColXV promoter (**D**) and intron 1 (**E**) in adipocytes were analysed after treating with 5-Aza-dC (*n* = 3). Data represent the mean ± SEM of three independent experiments. *$P < 0.05$, **$P < 0.01$.
